# Galactooligosaccharide Production Using Immobilized *Aspergillus oryzae* β-Galactosidase, Part I: Characterization and Influence of Reaction Conditions

**DOI:** 10.3390/ijms262311266

**Published:** 2025-11-21

**Authors:** Monika Antošová, Jana Krázel Adamíková, Milan Polakovič

**Affiliations:** Department of Chemical and Biochemical Engineering, Institute of Chemical and Environmental Engineering, Faculty of Chemical and Food Technology, Slovak University of Technology, Radlinského 9, 812 37 Bratislava, Slovakia; monika.antosova@stuba.sk (M.A.); adamikova.ja@gmail.com (J.K.A.)

**Keywords:** biocatalyst selectivity, enzyme immobilization, galactooligosaccharides, product yield, reaction kinetics, β-galactosidase

## Abstract

The enzymatic production of prebiotic galactooligosaccharides (GOS), functional food ingredients with established health benefits, remains an active research area driven by a rising global demand for GOS. These oligosaccharides are synthesized from lactose via transgalactosylation catalyzed by β-galactosidase, accompanied by hydrolysis of both substrate and products, and the competition between these reactions critically determines the maximum achievable GOS yield. In this study, β-galactosidase from *Aspergillus oryzae* was immobilized on an anion-exchange resin (Dowex Marathon MSA) using three glutaraldehyde-based crosslinking strategies. The resulting immobilized biocatalysts were characterized and evaluated for GOS synthesis, with product yield as the principal performance indicator. The results demonstrated that the immobilized biocatalysts markedly modulated the balance between transgalactosylation and hydrolytic activities. The biocatalyst prepared by simultaneous resin activation and enzyme crosslinking provided the highest GOS yield and operational stability. This biocatalyst was subsequently used to study the effects of lactose concentration, pH, enzyme loading, and temperature. Among these, lactose concentration most strongly influenced GOS yield, whereas the other factors primarily affected the reaction rate. These findings offer practical insights into enzyme immobilization strategies for optimizing GOS production.

## 1. Introduction

Galactooligosaccharides (GOS) are prebiotic oligosaccharides typically composed of one to five galactosyl units and a terminal glucose, linked by β(1→3)-, β(1→4)-, and β(1→6)-galactosidic bonds [1]. In addition to tri- and higher oligosaccharides, which typically have a degree of polymerization of up to six (DP6), the GOS group also includes disaccharides such as allolactose and galactobioses containing various glycosidic linkages between monosaccharides [2]. GOS are indigestible in the human gastrointestinal tract and are metabolized by the gut microbiota in the colon, thereby stimulating the growth of beneficial bacteria, particularly *Bifidobacterium* and *Lactobacillus* species.

This microbial activity also leads to the production of short-chain fatty acids, which lower the colonic pH [3] and promote various health benefits, such as reducing the risk of colon cancer, improving mineral absorption, and strengthening the immune system [1]. One study demonstrated up to a 44% reduction in the risk of atopic dermatitis in infants fed cow’s milk formula supplemented with a GOS and fructooligosaccharide blend, compared to a control group [4]. Therefore, the main application of GOS is in infant formula, but they are also used in other food industry sectors, such as the production of ice cream, yogurt, and breakfast cereals [5]. The growing consumer demand for prebiotics has led to a rapid expansion of the global GOS market, which is estimated to reach USD 1 billion in 2025, and is projected to grow to USD 2 billion by 2035, registering a solid compound annual growth rate (CAGR) of 7.5% [6].

GOS are produced by the transglycosylation of lactose catalyzed by β-galactosidase. β-Galactosidases are a group of enzymes that primarily catalyze the hydrolysis of lactose but also exhibit significant transglycosylation activity, enabling the formation of new glycosidic bonds. They are relatively common; enzymes derived from *Kluyveromyces lactis*, *Bacillus circulans*, *Aspergillus oryzae*, and *Bifidobacterium bifidum* are frequently employed for industrial purposes [7]. For instance, the enzyme from *B. circulans* predominantly forms β(1→4) glycosidic bonds [2], *B. bifidum* β-galactosidase forms β(1→2) and β(1→3) linked GOS [7], while β-galactosidases from *A. oryzae* and *K. lactis* mainly form β(1→6) and β(1→3) linkages in GOS [2,8]. Additionally, enzymes from different sources exhibit a varying ratio of transglycosylation to hydrolytic activity, which influences both the maximum yield and the composition of the resulting GOS [9,10].

The GOS yield is also influenced by reaction conditions such as pH and temperature, which affect enzyme activity and stability; therefore, it is essential to know their optimal values [11,12]. The presence of metal cations can either activate or inhibit the enzyme. For example, divalent Mg^2+^ cations are crucial for maintaining the activity of *K. lactis* β-galactosidase [13]. Another important parameter affecting the transglycosylation reaction is the initial lactose concentration [14]. Transgalactosylation is a kinetically controlled reaction in which lactose acts as both the donor and the acceptor of the galactosyl unit. Hydrolytic reactions of lactose and the formed GOS occur simultaneously, and the maximum GOS yield depends on the relative rates of the transglycosylation and competing hydrolytic reactions. High lactose concentrations favor GOS yield, as lactose serves as a more effective galactosyl donor and acceptor, while water activity is reduced in concentrated sugar solutions [14].

GOS synthesis can be performed using either free or immobilized enzymes. The advantage of using free enzymes lies in the simplicity of batch reactor operation and control, but it requires post-reaction enzyme deactivation and removal. On the other hand, the use of immobilized biocatalysts allows for easy separation of the enzyme from the product, their reuse in repeated cycles, or the possibility of continuous operation in packed-bed reactors [15].

The immobilization of β-galactosidase has been investigated using a wide range of techniques based on various types of enzyme–solid support interactions and employing different types of solid carriers. One approach involves the use of porous particles functionalized with ligands that enable covalent [16,17] or ion-exchange binding [18]. To prevent enzyme leaching from anion-exchange resins, immobilization is often accompanied by cross-linking with glutaraldehyde [16,19]. Both natural and synthetic polymers, such as chitosan [20] or silica [15,21,22], have also been investigated as solid supports. Another strategy involves entrapment or encapsulation of the enzyme within a polymeric matrix, such as alginate, gelatine, or polyvinyl alcohol, frequently combined with additional cross-linking [23,24,25]. Comprehensive overviews of β-galactosidase immobilization are provided in several reviews [26,27,28].

To further explore the benefits of enzyme immobilization for improved stability, reusability, and controlled operation, this study focused on evaluating GOS production using both free and immobilized β-galactosidase, with particular emphasis on the impact of immobilization on the reaction network, process stability, and biocatalyst performance. The enzyme source was a commercial powdered product containing β-galactosidase from *A. oryzae*. A strong anion-exchange resin was used for immobilization, with enzyme stabilization achieved by glutaraldehyde cross-linking using four different immobilization protocols [16,17]. The impact of the immobilization method on GOS formation was compared, and the optimal protocol was selected. Subsequently, the effects of reaction conditions—such as initial lactose concentration, temperature, pH, and enzyme amount in the reaction mixture—on GOS production were investigated.

## 2. Results and Discussion

**Figure 1:**
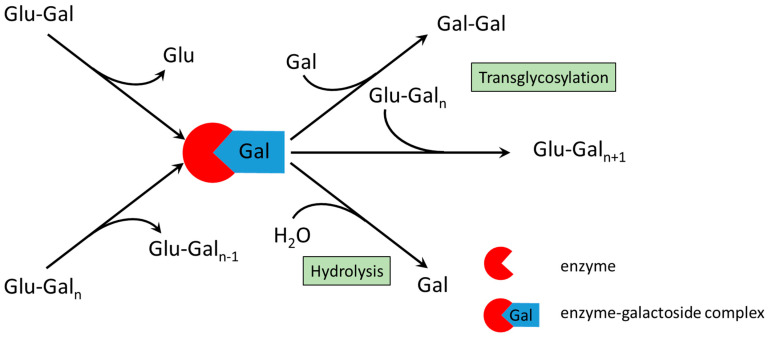
Reaction scheme of galactooligosaccharide synthesis by β-galactosidase. If n = 1 then Glu-Gal represents lactose; if n ≥ 2 then Glu-Gal_n_ represents a galactooligosaccharide; Gal-Gal denotes galactobiose.

To interpret the experimental results, a brief understanding of the reaction mechanism is necessary (Figure 1). The synthesis of GOS from lactose by β-galactosidase proceeds via a transglycosylation pathway involving two substrates. In the first step, lactose is cleaved to form an active enzyme–galactoside complex and free glucose. The intermediate then transfers the galactosyl residue to an acceptor molecule. When lactose serves as an acceptor, trisaccharides are formed; reaction with longer GOS yields higher oligosaccharides, while reactions with water leads to lactose hydrolysis into monosaccharides. Galactose itself can also act as an acceptor, forming galactobiose. Because oligosaccharides can function as both galactosyl donors and acceptors, the kinetically controlled system produces a complex mixture of GOS with varying chain lengths, along with residual lactose, glucose, and galactose (Figure 1).

A detailed explanation of the mechanism of kinetically controlled condensation reactions was provided by Kasche [29]. His conclusions indicate that in such reactions, the ratio of transferase to hydrolase activity depends on the intrinsic properties of the enzyme and substrate, as well as on the process conditions such as pH, temperature, ionic strength, and solvent composition. The catalytic behavior of an enzyme can also change upon immobilization, as reported by several authors and confirmed by our current and previous results [16].

### 2.1. Characterization of Free β-Galactosidase

**Figure 2:**
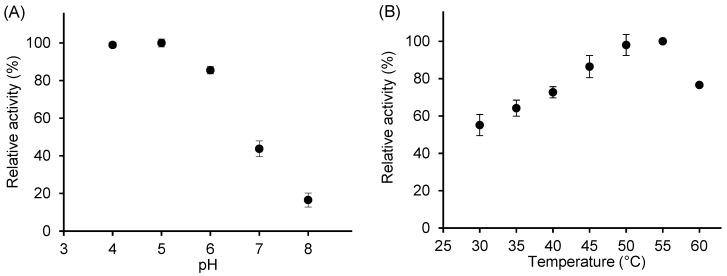
Effect of pH (**A**) and temperature (**B**) on the relative activity of free β-galactosidase. Reaction conditions: 13.2 mM ONPG; for pH effect, temperature was 42 °C; for temperature effect, pH was 4.5; enzyme concentration, 16.6 U/mL.

To ensure that the reaction proceeded under optimal conditions, the effects of temperature and pH on the activity of free β-galactosidase using o-nitrophenyl-β-D-galactopyranoside (ONPG) as the substrate were determined (Figure 2). The maximum activity was observed in the pH range of 4–5; therefore, pH 4.5 was selected for subsequent activity assays. Enzyme activity increased with temperature, reaching a maximum between 50 °C and 55 °C, and then decreased sharply (Figure 2B).

Our results of the effect of pH on *A. oryzae* β-galactosidase activity are consistent with those reported in the literature [30]. For lactose hydrolysis, the optimal pH was 4.5 with activity gradually decreasing at higher pH values; 50% of the activity was maintained at pH 8 and only 10% at pH 3 [17]. Similarly, Vera et al. [11] observed a broad optimum range of transgalactosylation activity between pH 2.5 and 5.5, followed by a sharp decline at higher pH. Minor variations among studies likely reflect differences in assay methods and substrates.

Our findings on the effect of temperature on β-galactosidase activity, showing an optimum in the range of 50–60 °C, are in an excellent agreement with previously published data [11,17,18]. However, a departure from the Arrhenius relationship of the initial activity is evident at the temperature of 50 °C. Above this temperature, thermal inactivation was thus demonstrated even during a short assay time. To safeguard enzyme stability in subsequent investigations, the GOS synthesis reactions were carried out at a lower temperature of 42 °C.

### 2.2. Characterization of Immobilized β-Galactosidase

The method of enzyme immobilization, along with the choice of carrier and immobilization conditions, determines the properties of the resulting biocatalyst. Based on prior work on β-galactosidase immobilization [16], a strong anion exchanger with a macroporous styrene-divinylbenzene matrix (Dowex Marathon MSA, average particle size 640 μm) was selected as the carrier. During immobilization, the negatively charged β-galactosidase binds ionically to the quaternary amine groups of the ion exchanger. This interaction can be weakened in the presence of salts, leading to possible enzyme desorption from the support [18]. Therefore, to enhance biocatalyst stability, three glutaraldehyde crosslinking strategies were employed [16].

The results of the immobilization experiments are summarized in Table 1. During immobilization, 40–50% of the enzyme were bound except for CAT2 (simultaneous resin activation and enzyme crosslinking), which showed an immobilization yield of only 20%. In the case of biocatalyst CAT2, a noticeable decrease in enzyme activity was observed when glutaraldehyde was mixed with the β-galactosidase solution prior to immobilization, suggesting that a fraction of the enzyme molecules became crosslinked and was unable to diffuse into the support particles. The immobilization yields expressed in terms of total protein binding did not differ significantly from those based on activity, indicating little or no activity loss during the immobilization.

However, the immobilization yields achieved in this work are within the range reported by other authors for similar types of supports. For example, β-galactosidase immobilization on the anion-exchange resin Purolite A109 resulted in a yield of around 40% [18]. In another study, β-galactosidase immobilization on Duolite A568, using a process similar to our protocol IV (adsorption followed by glutaraldehyde crosslinking), yielded 40.7% [19]. A much higher immobilization yield in terms of total protein, approximately 98%, was obtained on a quaternary ammonium agarose support, likely facilitated by the large pore size of 6% agarose beads compared to polymeric resins [31].

Interestingly, the activities of the prepared immobilized biocatalysts, *A_im_*, do not follow the trend of immobilization yield. CAT1 (without glutaraldehyde treatment) exhibited the highest activity, whereas all glutaraldehyde-stabilized biocatalysts showed lower activities. Biocatalyst CAT2, despite having the smallest amount of bound enzyme, displayed the third highest activity, while CAT4, prepared by post-crosslinking, showed the lowest. Although *A_im_* is a directly measured quantity, it should be regarded as an apparent value, since it reflects both the permanent loss of active enzyme during immobilization and the apparent loss of activity due to mass transfer limitations.

To investigate the effect of intraparticle diffusion on the overall reaction rate of ONPG hydrolysis, the Thiele modulus and effectiveness factor were calculated for all prepared biocatalysts (Table 1). Detailed information on the calculation methods is provided in our previous publication by Adamíková et al. [16]. The Thiele modulus values ranged from 7.7 to 16.3, which implies that the reaction rate was limited by intraparticle mass transfer, which resulted in low effectiveness factors. The main causes of the low effectiveness factors were the relatively large particle diameter, high intraparticle enzyme concentration, and non-uniform enzyme distribution [16]. As discussed in our previous work, the use of biocatalysts with lower effectiveness factors also offers several advantages, including higher volumetric productivity, lower pressure drop, and longer biocatalyst lifetime. However, it should be noted that diffusion effects on biocatalyst performance are much less pronounced at the high lactose concentrations employed in industrial production [32].

**Figure 3:**
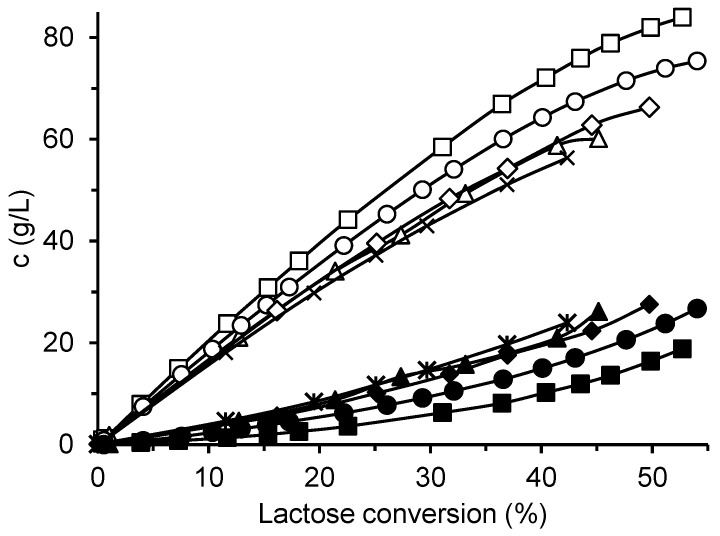
Production of GOS and galactose by free and immobilized β-galactosidase. Reaction conditions: initial lactose concentration 300 g/L; pH 4.5; temperature 42 °C; free enzyme concentration 16.6 U/mL; mass of biocatalyst 80 mg per 1 mL of lactose solution. Symbols: free enzyme (GOS □, Gal ■); immobilized biocatalysts CAT1 (GOS △, Gal ▲), CAT2 (GOS ◯, Gal ●), CAT3 (GOS 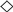, Gal ◆), and CAT4 (GOS ✕, Gal 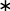).

The four prepared immobilized biocatalysts were used for GOS synthesis from lactose, and their catalytic properties were compared with those of the free enzyme. Figure 3 shows the production of total GOS and galactose by the various enzyme forms using an initial lactose concentration of 300 g/L. The free enzyme produced the highest amount of GOS (up to 84 g/L) and, at the same time, the lowest amount of galactose. All immobilized biocatalysts exhibited higher hydrolytic activity at the expense of transferase activity, which resulted in lower GOS and higher galactose formation. Among the immobilized biocatalysts, CAT2 showed the best performance, forming 75 g/L of GOS. Interestingly, this biocatalyst, prepared by dissolving the enzyme in a glutaraldehyde-buffer solution, did not exhibit the highest activity toward the ONPG substrate. The biocatalyst CAT4, which had the lowest ONPG activity and was prepared by enzyme adsorption followed by additional glutaraldehyde crosslinking, displayed the poorest GOS-to-galactose ratio.

Enzyme immobilization, as well as the glutaraldehyde crosslinking method, evidently altered the ratio between the transgalactosylation and hydrolytic activities of β-galactosidase, thereby affecting the GOS yield. Although many authors have reported no change in β-galactosidase selectivity upon immobilization [33,34,35], others have observed significant differences manifested as either a decrease [12,36] or increase in GOS yield [12,18,22,24,37]. Guerrero et al. reported both positive and negative effects of different *A. oryzae* β-galactosidase immobilization methods on GOS yield and on the profiles of individual GOS [38]. They attributed these variations to differences in enzyme orientation resulting from the immobilization method, combined with varying degrees of diffusional restriction, which together modified enzyme selectivity.

To the best of our knowledge, there are two possible explanations for the lower GOS yield obtained with the immobilized biocatalysts in this study. The first assumes that modifications in the tertiary structure or spatial orientation of the bound enzyme may impair the accessibility of its active site for the galactosyl acceptor. The second considers the formation of substrate and product concentration gradients within the particles due to mass transfer limitations, leading to a low local lactose concentration and high local GOS concentration, which in turn increases the probability of GOS hydrolysis [16]. However, an interaction between these two phenomena cannot be excluded.

Although a higher GOS yield was achieved with the free enzyme, the advantages of immobilized systems prompted further investigation of their properties and potential applications. A known limitation of enzyme immobilization on ion-exchange resins is the possible loss of activity due to enzyme leaching, caused by changes in pH or ionic strength during the reaction. To address this issue, the biocatalysts CAT2, CAT3, and CAT4 were stabilized by glutaraldehyde crosslinking, and their stability was compared with that of the non-stabilized CAT1. For this purpose, six consecutive batch reaction cycles were carried out under standard reaction GOS synthesis conditions.

**Figure 4:**
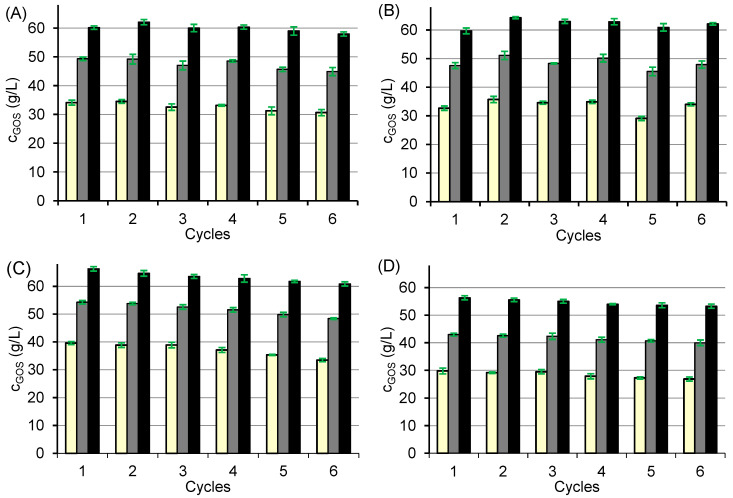
Stability of biocatalysts CAT1 (**A**), CAT2 (**B**), CAT3 (**C**), and CAT4 (**D**) over six consecutive reaction cycles. Reaction conditions: initial lactose concentration 300 g/L; pH 4.5; temperature 42 °C; biocatalyst mass 80 mg per 1 mL of lactose solution; reaction times of 30 min (light yellow), 60 min (grey), and 120 min (black).

The results (Figure 4) show that none of the biocatalysts reached the maximum GOS concentration within the tested time frame, yet the data clearly demonstrate the production dynamics and operational stability of each biocatalyst. The GOS concentration achieved per cycle was approximately 60 g/L for CAT1–CAT3, while CAT4 produced somewhat less due to its higher hydrolytic activity (Figure 3). Among the tested systems, CAT2 showed the best operational stability (Figure 4B), maintaining GOS production of at least 60 g/L in all six cycles. In contrast, CAT1, CAT3, and CAT4 exhibited a gradual decline in GOS yield by about 7–10% over successive cycles, reaching about 90–93% of the initial value after the 6th cycle (Figure 4A,C,D).

For CAT1, where the enzyme is bound solely by ionic interactions, this decrease likely resulted from enzyme leaching. Conditioning the carrier with glutaraldehyde before immobilization (CAT3) or additional post-immobilization crosslinking (CAT4) did not improve stability, suggesting that the observed loss of activity was primarily due to the gradual enzyme inactivation rather than leaching.

Our results on biocatalyst stability are consistent with previously published findings on similar immobilized β-galactosidases. For example, *A. oryzae* β-galactosidase immobilized on Duolite A568 and cross-linked with glutaraldehyde retained 90% of its initial activity after 30 cycles, whereas the non-cross-linked counterpart exhibited a decrease in activity to 51% [19]. Using a modified three-step immobilization procedure on the same resin, even higher stability was achieved, with lactose conversion remaining constant over 10 consecutive cycles [39]. In another study, the GOS yield obtained with *A. oryzae* β-galactosidase immobilized on SiO_2_ nanoparticles showed no significant change over five cycles [15].

By contrast, Hackenhaar et al. reported a gradual decline in GOS production using *B. circulans* β-galactosidase immobilized on glutaraldehyde-activated chitosan [20]. Similarly, a 10% decrease in the activity of β-galactosidase immobilized on a methacrylate resin bearing primary amine groups was observed after four reaction cycles, although GOS production efficiency remained almost unchanged [40]. Collectively, these findings indicate that the CAT2 biocatalyst exhibits superior operational stability and is particularly well-suited for GOS production. Therefore, CAT2 was selected for further studies on GOS synthesis.

### 2.3. Effect of Reaction Conditions on GOS Synthesis

As described in the introduction, the production of GOS from lactose involves a kinetically controlled system of two-substrate transglycosylation reactions. Because the reaction rate depends on the reaction conditions, it is essential to investigate the effects of initial lactose concentration (ILC), enzyme amount, pH, and temperature on the GOS formation. In this study, a pH of 5, temperature of 42 °C, and ILC of 300 g/L were selected as the standard reaction conditions. The influence of ILC, varied from 10 to 300 g/L, was examined for both the free enzyme and the immobilized biocatalyst CAT2. Although supersaturated lactose solutions can provide higher GOS yields [14], the limited lactose solubility of 33.5 g per 100 g of water at 40 °C [41] increases the risk of solution instability and lactose crystallization during the process. Therefore, the ILC was not increased beyond 300 g/L.

**Figure 5:**
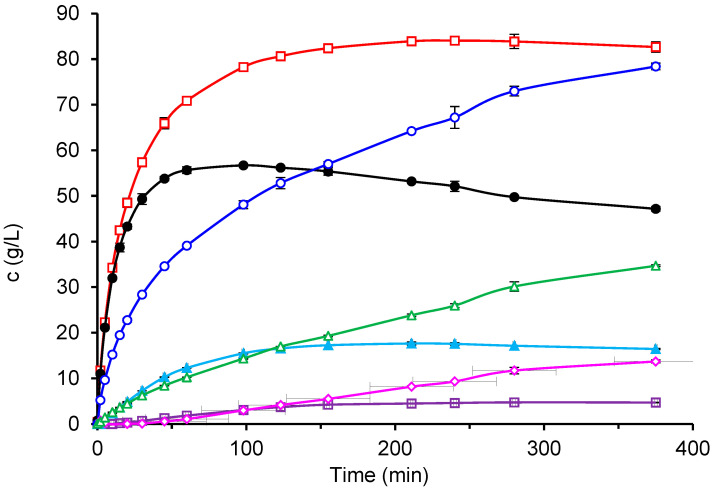
Course of formation of individual carbohydrates during the reaction catalyzed by free β-galactosidase at ILC of 300 g/L, pH 4.5, temperature 42 °C, and enzyme concentration 33.3 U/mL. Symbols: 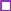 GOS5, 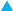 GOS4, ● GOS3, 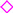 Glb, 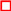 total GOS, 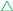 Gal and 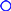 Glu (Lines are provided for eye guidance only).

An example of the reaction course with the free enzyme at an ILC of 300 g/L is shown in Figure 5. The trisaccharide GOS3 formed rapidly at the beginning of the reaction, reached its maximum concentration, and was then depleted as GOS4 synthesis proceeded. As the reaction progressed, longer-chain oligosaccharides up to GOS6 were detected in small amounts. In contrast, no GOS6 formation was observed with the immobilized biocatalyst CAT2, although the overall GOS composition was similar to that obtained with the free enzyme. In both cases, galactobiose was produced in substantial amounts, increasing with reaction time. The total GOS concentration reached a maximum at a certain lactose conversion and subsequently decreased due to hydrolysis. The hydrolytic activity of the enzyme was also evident from the substantial amount of galactose formed (empty triangles in Figure 5).

**Figure 6:**
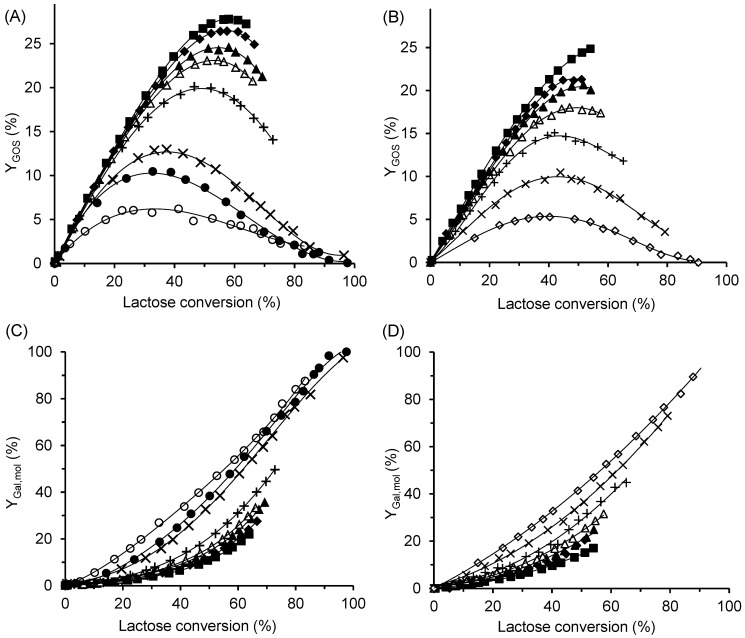
Effect of initial lactose concentration on GOS production (**A**,**B**) and lactose hydrolysis (**C**,**D**). The enzyme was either free β-galactosidase (**A**,**C**), or immobilized biocatalyst CAT2 (**B**,**D**). ILC values: ■ 300 g/L, ◆ 250 g/L, ▲ 200 g/L, △ 150 g/L, + 100 g/L, ✕ 50 g/L, ● 30 g g/L, 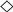 25 g/L, and ◯ 10 g/L. Reaction conditions: temperature 42 °C, pH 4.5, enzyme concentration 16.7 U/mL (free enzyme) and 4.5 U/mL (CAT2). The percentage of hydrolyzed lactose was calculated as the ratio of galactose formed to the initial lactose concentration.

The effect of ILC on the GOS yield, $Y_{GOS}$, is shown in Figure 6A,B, while the time courses of total GOS formation at different ILC values are provided in Figure S2 in the Supplementary Material. Increasing the ILC had a positive effect on both GOS yield and the total amount of GOS produced by the free enzyme (Figure 6A) and CAT2 (Figure 6B). At all lactose concentrations tested, the free enzyme produced a higher maximum amount of GOS than CAT2. The highest GOS yields—almost 28% for the free enzyme and approximately 25% for CAT2—were obtained at an ILC of 300 g/L, corresponding to total GOS concentrations of 85 g/L and 75 g/L, respectively. The lactose conversion at which the maximum yield occurred increased with increasing ILC, regardless of the enzyme form. At conversions approaching 100%, both GOS and lactose were completely hydrolyzed to monosaccharides. Nevertheless, even at the lowest lactose concentration of 10 g/L, oligosaccharides were formed, reaching a maximum yield of 6%, which confirms the transgalactosylation ability of the enzyme.

The hydrolytic activity of the enzyme correlates with the amount of free galactose, as shown in Figure 1. To evaluate the effect of ILC, the molar yield of galactose, $Y_{Gal,mol}$, was calculated as the ratio of the molar concentration of galactose to the initial molar concentration of lactose. As shown in Figure 6C,D, hydrolysis was markedly suppressed at high ILC values (200–300 g/L) for both the free and immobilized enzymes. In contrast, at ILC values below 30 g/L, the curves approached the diagonal, indicating that most of the consumed lactose was hydrolyzed to monosaccharides.

The initial lactose concentration also strongly affected the relative proportions of individual GOS in the product mixture, as shown in Figure S3 and Tables S1 and S2 in the Supplementary Material. At low lactose concentrations, trisaccharides were the predominant GOS, whereas increasing the lactose concentration favored the formation of higher oligosaccharides up to GOS5, as well as galactobiose. Although the total amount of GOS obtained with the immobilized enzyme was lower, the composition of the oligosaccharide mixture was comparable to that produced by the free enzyme.

It has been well established that increasing the initial lactose concentration shifts the reaction toward higher GOS yields [42]. This effect arises because lactose serves as both a galactosyl donor and acceptor, while the concomitant reduction in water activity significantly suppresses hydrolysis. The maximum GOS yields obtained in our study with free *A. oryzae* β-galactosidase are comparable to those reported by other authors using the same enzyme. For instance, Cinar et al. [43] achieved a GOS yield of 27% at an ILC of 32° Brix. Vera et al. [14] reported a maximum total GOS concentration of 185 g/L using a supersaturated lactose solution of 50% (*w*/*w*), corresponding to a yield of 29%, whereas Wang et al. [44] obtained an optimum yield of GOS of 25.1% at an ILC of 50% (*w*/*v*).

In our case, the maximum GOS yield was approximately 3% lower when using the immobilized biocatalyst, which can be attributed to the enhanced hydrolytic activity of CAT2. Nevertheless, this yield remained higher than the 20% obtained in a batch reactor with β-galactosidase immobilized on the ion exchanger Purolite A109 at an ILC of 400 g/L [18]. Overall, the total GOS yield of 25% achieved in this study can still be considered economically viable.

**Figure 7:**
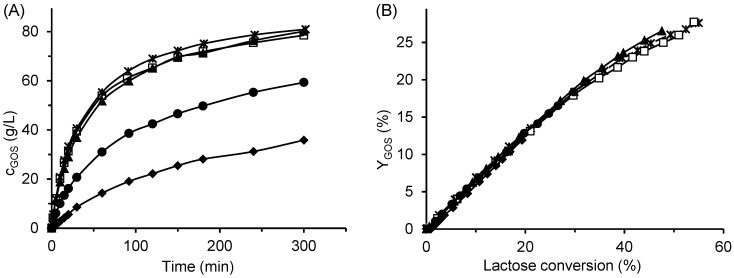
Effect of pH on GOS production. (**A**) Time course of GOS concentration and (**B**) GOS yield dependence of GOS yield on lactose conversion. Symbols: 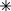 pH 4, □ pH 5, ▲ pH 6, ● pH 7, and ◆ pH 8. Reaction conditions: initial lactose concentration 300 g/L, enzyme concentration 16.6 U/mL (free enzyme), temperature 42 °C.

Enzyme-substrate interactions are closely linked to the enzyme’s three-dimensional structure, which determines the conformation of the active site and, consequently, the enzyme’s affinity and specificity toward the substrate. Among various factors, pH plays a critical role in maintaining the protein structure and can influence the balance between transgalactosylation and hydrolytic activities. Therefore, despite the known pH optimum for ONPG hydrolysis, we examined the effect of pH on GOS formation. The results are shown in Figure 7. Within the pH range of 4–6, no significant differences were observed in either the total amount of GOS produced or the reaction rate (Figure 7A). Although the reaction rate decreased at pH values between 6 and 8, the GOS yield at a given lactose conversion remained unchanged (Figure 7B), indicating that the ratio of transglycosylation to hydrolytic activity was not affected by pH. Likewise, the concentrations of individual oligosaccharides did not differ at the same lactose conversion (Figure S4, Supplementary Material). Similar trends have also been reported by other authors using *A. oryzae* β-galactosidase [11,25,33,35,45].

In contrast to the approximately 20% decrease in enzyme activity toward the ONPG substrate observed when the pH increased from 5 to 6, GOS production was not affected by this pH change. We attribute this behaviour to the stabilizing effect of carbohydrates—particularly lactose, and likely also GOS—on the enzyme structure. This interpretation is supported by our previous study, in which the thermal stability of β-galactosidase was enhanced in the presence of lactose [46].

Another important parameter influencing enzyme-catalyzed reactions is the enzyme concentration in the reaction mixture. Increasing the enzyme amount in the reaction accelerates the reaction rate proportionally, thereby shortening the reaction time and improving reactor volumetric productivity. However, several authors have reported that excessive enzyme concentrations can enhance hydrolysis and consequently decrease GOS yield [47,48]. From an economic point of standpoint, the enzyme cost often represents a major component of total production expenses. Therefore, an optimal enzyme concentration must balance productivity and cost to minimize the overall production expense. In this study, the effect of free enzyme concentration in the range of 3.3–33.3 U/mL on the kinetics and yield of GOS formation was investigated.

**Figure 8:**
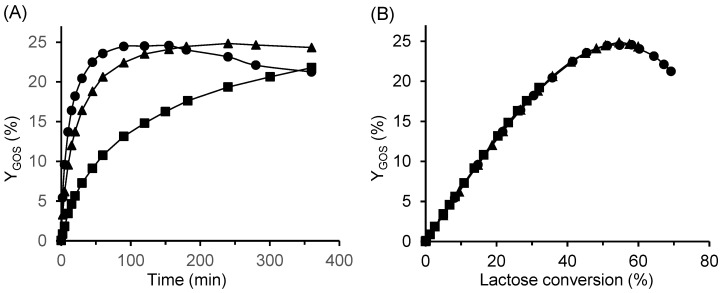
Effect of enzyme concentration on GOS yield as a function of (**A**) time and (**B**) lactose conversion. Reaction conditions: initial lactose concentration 200 g/L, pH 4.5, temperature 42 °C; free enzyme concentrations: ■ 3.3 U/mL, ▲ 16.7 U/mL, and ● 33.3 U/mL.

The results obtained are presented in Figure 8. Enzyme concentration had a pronounced effect on the rate of GOS production. At the lowest enzyme concentration, the reaction proceeded slowly, and the maximum GOS yield was not reached within 6 h. In contrast, at the highest concentration (33.3 U/mL), the maximum yield was achieved within 120 min, followed by a gradual decrease (Figure 8A). However, the total amount of GOS produced at a given lactose conversion was identical regardless of the enzyme concentration used (Figure 8B). No change was observed in the relative composition of individual GOS species (Figure S5 in the Supplementary Material). Consequently, the volumetric productivity at the point of maximum yield increased from 12.5 to 24.8 g/L/h as the enzyme concentration doubled from 16.7 to 33.3 U/mL. A similar reaction pattern was also observed for reactions catalyzed by the immobilized β-galactosidase CAT2.

Because enzyme concentration proportionally accelerates all concurrent reactions in the scheme shown in Figure 1, it influences only the overall reaction rate but not the composition of the product mixture at a given conversion. Similar behavior in GOS synthesis has been reported by several authors [35,49,50]. Our findings also agree with the fundamental work of Kasche [29], who demonstrated that in kinetically controlled syntheses catalyzed by hydrolases, the maximum yield depends on the intrinsic properties of the enzyme and reaction system as well as on the type of substrate activation, whereas enzyme concentration affects only the rate at which this maximum is reached. The system parameters governing the maximum yield include pH, ionic strength, temperature, and solvent composition.

Temperature exerts an ambivalent effect on enzymatic reactions: increasing temperature enhances the catalytic rate constant *k_cat_* but simultaneously destabilizes the protein structure, leading to enzyme inactivation. Moreover, temperature influences product yield in kinetically controlled reactions [29]. The temperature optimum for enzyme activity is therefore not identical to that for the overall process. Consequently, it is essential to examine the effect of temperature not only on GOS yield but also on the stability and operational lifetime of the biocatalyst.

**Figure 9:**
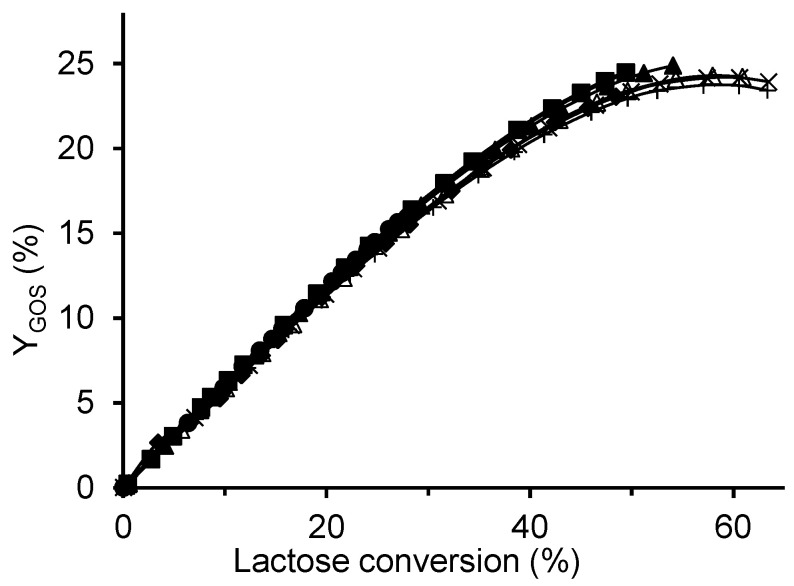
Effect of temperature on GOS yield using the CAT2 biocatalyst. Reaction conditions: initial lactose concentration 300 g/L, enzyme concentration 4 U/mL, pH 4.5; temperatures: ■ 30 °C, ◆ 35 °C, ▲ 40 °C, △ 50 °C, + 57.5 °C, ✕ 60 °C, and ● 66.5 °C.

Figure 9 shows that the reaction temperature had only a minor effect on GOS yield, as all curves were nearly identical. The highest maximum yield of 25% was obtained at the lowest temperature (30 °C), compared to 24% at 60 °C. Similar observations in the temperature range of 30–60 °C were reported by Albayrak et al. [33] and Neri et al. [25]. The slightly higher total GOS yield at lower temperatures resulted mainly from increased formation of tri- and tetrasaccharides, and simultaneously, a reduced amount of galactose (Figure S6 in Supplementary Material), indicating that lower temperatures favored the transglycosylation activity of the enzyme at the expense of its hydrolytic activity. However, this effect was relatively minor. In addition, Figure 9 demonstrates a nearly linear correlation between GOS yield and lactose conversion at conversions below 30%.

Because temperature simultaneously affects enzyme activity, stability, and thus overall process efficiency, understanding its influence is essential for optimizing biocatalyst performance. A detailed analysis of the temperature impact on the productivity and operational stability of the immobilized biocatalyst, together with an assessment of the resulting economic implications and potential strategies for process optimization will be presented in the second part of this study.

## 3. Materials and Methods

### 3.1. Materials

Anion-exchange resin Dowex Marathon MSA was purchased from Dow Chemical Company (Midland, TX, USA). β-Galactosidase powder Tegaferm LAC A100P from *A. oryzae* with declared activity of 100,000 U/g was obtained from Tegaferm Holding GmbH (Wien, Austria). Glutaraldehyde, ONPG, and 2-nitrophenol were supplied by Sigma-Aldrich (St. Louis, MO, USA). Lactose was obtained from Centralchem (Bratislava, Slovakia). All other chemicals used were of analytical grade and available commercially.

### 3.2. β-Galactosidase Immobilization

Immobilization methods were adopted from Adamíková et al. [16]. The resin was first washed with distilled water and then equilibrated with a 100 mM citrate-phosphate buffer (pH 7), hereafter referred to as the immobilization buffer. Four different protocols of immobilization were employed:

#### 3.2.1. Protocol I

β-galactosidase solution was prepared by dissolving powdered β-galactosidase in the immobilization buffer at the concentration of 5 g/L. One gram of wet resin was added to 5 mL of enzyme solution and incubated in an orbital shaker (GFL, Burgwedel, Germany) at ambient temperature and agitated at 25 rpm for 6 h.

#### 3.2.2. Protocol II

Glutaraldehyde was added to the immobilization buffer at the concentration of 1% (*w*/*v*) before enzyme dissolution. All other conditions were identical to those in Protocol I.

#### 3.2.3. Protocol III

One gram of resin was pre-activated before contact with the enzyme solution with 5 mL of immobilization buffer containing 1% (*w*/*v*) of glutaraldehyde at room temperature under agitation for 3 h. The excess glutaraldehyde was removed by washing with immobilization buffer and β-galactosidase immobilization was performed according to Protocol I.

#### 3.2.4. Protocol IV

β-galactosidase was immobilized onto a resin following Protocol I. The immobilized enzyme was then crosslinked by glutaraldehyde. The resin with the immobilized enzyme was mixed with 5 mL of the immobilization buffer containing 1% (*w*/*v*) of glutaraldehyde at room temperature under agitation for 3 h.

After immobilization, the particles of immobilized biocatalyst were washed in three steps. They were first washed with an immobilization buffer, then with a 10 mM acetate buffer (pH 5.5) and finally with redistilled water. All biocatalysts were prepared at least in duplicate.

Immobilization yield, $Y_{im}$, and immobilization yield in terms of total protein binding, $Y_{im,prot}$, were calculated as a ratio of enzyme bound to the particle to initial enzyme amount.

(1) $$Y_{im}=\frac{A_{0}-A}{A_{0}}$$

(2) $$Y_{im,prot}=\frac{c_{p,0}-c_{p}}{c_{p,0}}$$

where *A*_0_ and *A* are enzyme activities, *c_P_*_,0_ and *c_P_* are protein concentrations before and after immobilization, respectively. Thiele modulus and effectiveness factor were calculated by method in Adamíková et al. [16].

### 3.3. Production of Galactooligosaccharides

Galactooligosaccharides were produced in batch reactors with a working volume of 20 mL agitated with magnetic stirrer at frequency of 220 rpm. Standard reaction conditions were initial lactose concentration of 300 g/L (in 100 mM acetate buffer with pH 5) and temperature of 42 °C. These conditions were appropriately modified in studies investigating the effects of pH, temperature and substrate concentration effects. Buffers used in experiments with different pH were 100 mM acetate buffer for pH 4 and 5, and 100 mM sodium phosphate buffer for pH 6–8. In substrate concentration effect study solutions with initial lactose concentration in the range 10–300 g/L were prepared by dissolving lactose in 100 mM acetate buffer pH 5. Stock solution of free enzyme was prepared by dissolving β-galactosidase powder in saline solution at concentration of 4 g/L (700 U/mL). 1 mL of appropriate diluted enzyme was added into 20 mL of substrate solution preheated to the reaction temperature. In the case of immobilized enzyme, 40–45 mg of wet biocatalyst per 1 mL of substrate solution was used. Samples of 50 μL were withdrawn from the reactor at selected time intervals and immediately mixed with 30 μL of 100 mM NaOH to stop the reaction. All experiments were conducted in duplicate. Values reported are the corresponding averages and the mean errors were less than 2%.

GOS yield was calculated as the ratio of total GOS concentration, $c_{GOS}$, to the initial lactose concentration, $c_{Lac,0}$

(3) $$Y_{GOS}=\frac{c_{GOS}}{c_{Lac,0}}$$

Content of an individual oligosaccharide in total GOS was calculated as the mass fraction


(4) $$w_{GOS,i}=\frac{c_{GOS,i}}{c_{GOS}}$$

Lactose conversion was calculated as

(5) $$X=\frac{c_{Lac,0}-c_{Lac}}{c_{Lac,0}}$$

### 3.4. β-Galactosidase Activity Assay

β-Galactosidase activity was determined using ONPG as a substrate at 42 °C [16]. 4.5 mL of 12.3 mM ONPG solution in 100 mM acetate buffer with pH 4.5 was mixed with 20 µL of the enzyme solution. For the determination of immobilized β-galactosidase activity, 40 mg of wet biocatalyst was added to 20 mL of ONPG solution. In case of pH effect determination, 100 mM sodium phosphate buffer was used for pH’s 6–8. At predefined time intervals, samples of 550 µL were withdrawn and immediately mixed with 550 µL of a 10% (*w*/*v*) sodium carbonate solution to stop the reaction. The amount of o-nitrophenol released was measured spectrophotometrically at 420 nm on an Agilent Cary 100 (Santa Clara, CA, USA). One Unit was defined as the amount of the enzyme required to hydrolyze 1.0 µmol of ONPG per minute at pH 4.5 and 42 °C. All activity assays were performed in triplicates and the error expressed as the standard deviation of three measurements was less than 7%. The determined activity of Tegaferm enzyme was 174.0 ± 3.1 U/mg of enzyme powder.

### 3.5. Protein Assay

The protein concentration in enzyme solutions was determined by linearized Bradford method [51] using bovine serum albumin as the standard. The protein content in the Tegaferm LAC A100P powder was 56% (*w*/*w*).

### 3.6. HPLC Analyses

The saccharide content in the samples was analyzed by Agilent 1200 HPLC with refractometric detection (Santa Clara, CA, USA) using a BP-200 Ag column with dimensions of 300 mm × 7.8 mm (Benson Polymeric, Reno, NV, USA) operated at 80 °C. Redistilled water with the flow rate of 0.4 mL/min was used as a mobile phase. The injection volume was 10 μL and RI detector was maintained at 30 °C.

Peak identification was based on comparison of retention times of standard peaks of lactose, galactose and glucose (Sigma-Aldrich, St. Louis, MO, USA) or peaks obtained by analysis of Vivinal^®^ GOS syrup (Friesland Campina, Amersfoort, The Netherlands). Quantification of glucose and galactose was performed using the external standard method, quantification of oligosaccharides GOS3–GOS6 was based on peak area percentage. The BP-200 Ag column lacks the selectivity to separate lactose from other disaccharides and disaccharides coeluted in one peak at 18.44 min with partially resolved back shoulder (Figure S1 in Supplementary Material). It is well known that *A. oryzae* β-galactosidase catalyses formation of various disaccharides from lactose, with the majority of allolactose and 6-galactobiose or 4-galactobiose [7,8,52,53]. A simple way to confirm of galactobiose formation (regardless of the type of glycosilic bond) is to calculate the proportion of amount glucosyl and galactosyl residues from the mass balance, assuming that all di- and oligosaccharides contain one glucosyl [54,55]. If only oligosaccharides containing one glucosyl are formed, the ratio of molar concentrations of total glucosyl to galactosyl moieties must be equal to 1. In our experiments, the ratio was greater than 1 with an increasing value as the reaction time increased, indicating that galactobiose was formed during the reaction. The material balances of the total glucosyl and galactosyl residues assuming only disaccharide galactobiose (Glb) is formed are then in the form:

(6) $$c_{Gal,tot}=c_{Lac,ini}=c_{Gal}+c_{Lac}+2c_{Glb}+2c_{GOS3}+3c_{GOS4}+4c_{GOS5}+5c_{GOS6}$$

(7) $$c_{Glu,tot}=c_{Lac,ini}=c_{Glu}+c_{Lac}+c_{GOS3}+c_{GOS4}+c_{GOS5}+c_{GOS6}$$

Transforming Equations (6) and (7), the corrected concentrations of lactose and galactobiose were calculated:

(8) $$c_{Glb}=0.5\left(c_{Glu}-c_{Gal}-c_{GOS3}-2c_{GOS4}-3c_{GOS5}-4c_{GOS6}\right)$$

(9) $$c_{Lac}=c_{Lac,ini}-c_{Glu}-c_{GOS3}-c_{GOS4}-c_{GOS5}-c_{GOS6}$$

In this work, the total GOS are considered as a mixture of galactooligosaccharides GOS3–GOS6 as well as disaccharide galactobiose since its physiological characteristics are similar to those of longer chains oligosaccharides [56].

## 4. Conclusions

This study demonstrated that the method of immobilization and enzyme stabilization using glutaraldehyde strongly influences the balance between the hydrolytic and transgalactosylation activities of the resulting biocatalysts. Among the tested preparations, the most stable and productive biocatalyst—with the most favorable transgalactosylation-to-hydrolysis activity ratio—was obtained by simultaneous carrier activation and enzyme crosslinking with glutaraldehyde.

GOS production using this biocatalyst was most affected by the initial lactose concentration. High lactose concentrations substantially suppressed hydrolytic degradation, resulting in the highest maximum GOS yield, whereas, at low lactose concentrations, enhanced hydrolysis of both GOS and lactose to monosaccharides was observed. pH and enzyme loading primarily influenced the reaction rate, although these parameters had little effect on the GOS yield or product composition. While higher enzyme loading shortened reaction time, it would also increase production costs, indicating the need for further economic optimization.

Temperature was identified as another key factor in GOS synthesis. Although a slight decrease in maximum GOS yield was observed at elevated temperatures, temperature also affected both the reaction rate and the stability of the biocatalyst. A comprehensive evaluation of temperature effects on GOS production—including kinetic behavior, long-term operational stability of the CAT2 biocatalyst, and the economic feasibility of industrial-scale GOS production using both free and immobilized enzymes—will be presented in Part II of this study. Overall, the present work provides fundamental insights into how immobilization strategy and reaction conditions determine the catalytic balance and process efficiency of β-galactosidase, establishing a robust framework for optimizing enzymatic GOS synthesis in industrial applications.

## Figures and Tables

**Table 1:** Characteristics of biocatalysts prepared either without glutaraldehyde treatment (CAT1) or with glutaraldehyde treatment (CAT2–CAT4) using the corresponding Protocols I–IV specified in Section 3.2 [16].

	$A_{im}$ (U/g)	$Y_{im}$ (%)	$Y_{im,prot}$ (%)	Thiele Modulus *	Effectiveness Factor
CAT1	148.5 ± 1.1	44.4 ± 0.2	43.4 ± 2.1	16.3	0.23
CAT2	89.1 ± 1.6	19.8 ± 1.0	23.5 ± 1.3	10.2	0.36
CAT3	102.0 ± 3.1	40.9 ± 1.1	48.8 ± 0.6	11.5	0.32
CAT4	65.0 ± 1.2	49.1 ± 0.3	52.3 ± 1.2	7.7	0.46

* The uncertainties in the Thiele modulus values were up to 20% due to the estimated effective diffusion coefficient of ONPG [16].

## Data Availability

The original contributions presented in this study are included in the article/Supplementary Material. Further inquiries can be directed to the corresponding author.

## Supplementary Materials

The supporting information can be downloaded at: https://www.mdpi.com/article/10.3390/ijms262311266/s1.
