# Supplementary material for: Galactooligosaccharide Production Using Immobilized Aspergillus oryzae β-Galactosidase, Part I: Characterization and Influence of Reaction Conditions
Source: Int J Mol Sci. 2025 Nov 21;26(23):11266. doi: 10.3390/ijms262311266 (PMC12692611; doi:10.3390/ijms262311266)
Supplement: Supplementary file 1 [file ijms-26-11266-s001.zip › ijms-3992625-supplementary.pdf]

Supplementary Material

Galactooligosaccharide production using immobilized Aspergillus oryzae β-galactosidase: Part I. Characterization and influence of reaction conditions

Monika Antošová, Jana Krázel Adamíková, Milan Polakovič*

Department of Chemical and Biochemical Engineering Institute of Chemical and Environmental Engineering, Faculty of Chemical and Food Technology, Slovak University of Technology, Radlinského 9, 812 37 Bratislava, Slovakia

* Correspondence: milan.polakovic@stuba.sk

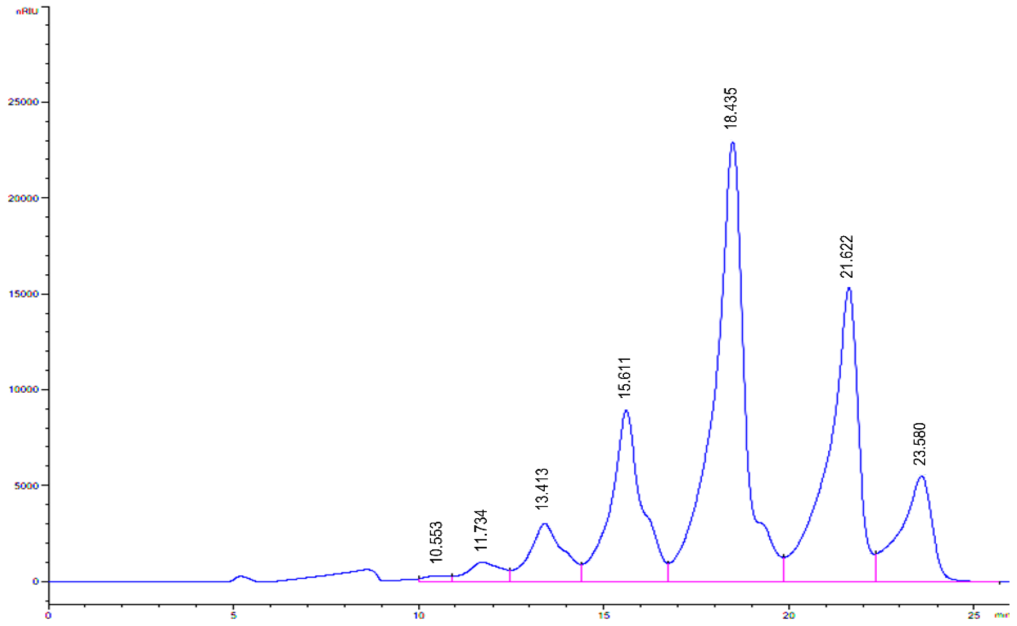

Figure S1. Illustrative HPLC chromatogram of the reaction mixture. Peak identification according to retention time (min): GOS6 (10.53), GOS5 (11.73), GOS4 (13.41), GOS3 (15.61), lactose and disaccharides (18.44), glucose (21.62), and galactose (23.58).

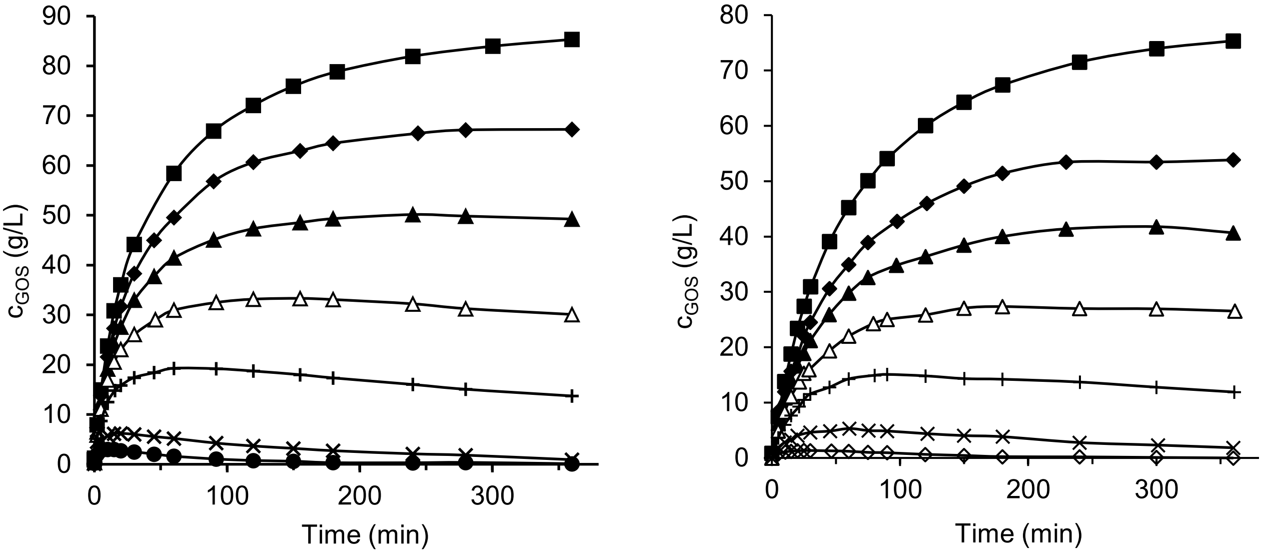

Figure S2. Effect of initial lactose concentration on GOS production catalyzed by free β-galactosidase (A), or immobilized biocatalyst CAT2 (B). Initial lactose concentrations: ■ 300 g/L, ◆ 250 g/L, ▲ 200 g/L, △ 150 g/L, + 100 g/L, × 50 g/L, ● 30 g g/L, and ◇ 25 g/L. Reaction conditions: temperature 42°C, enzyme concentration 16.7 U/mL (free enzyme) and 4.5 U/mL (CAT2).

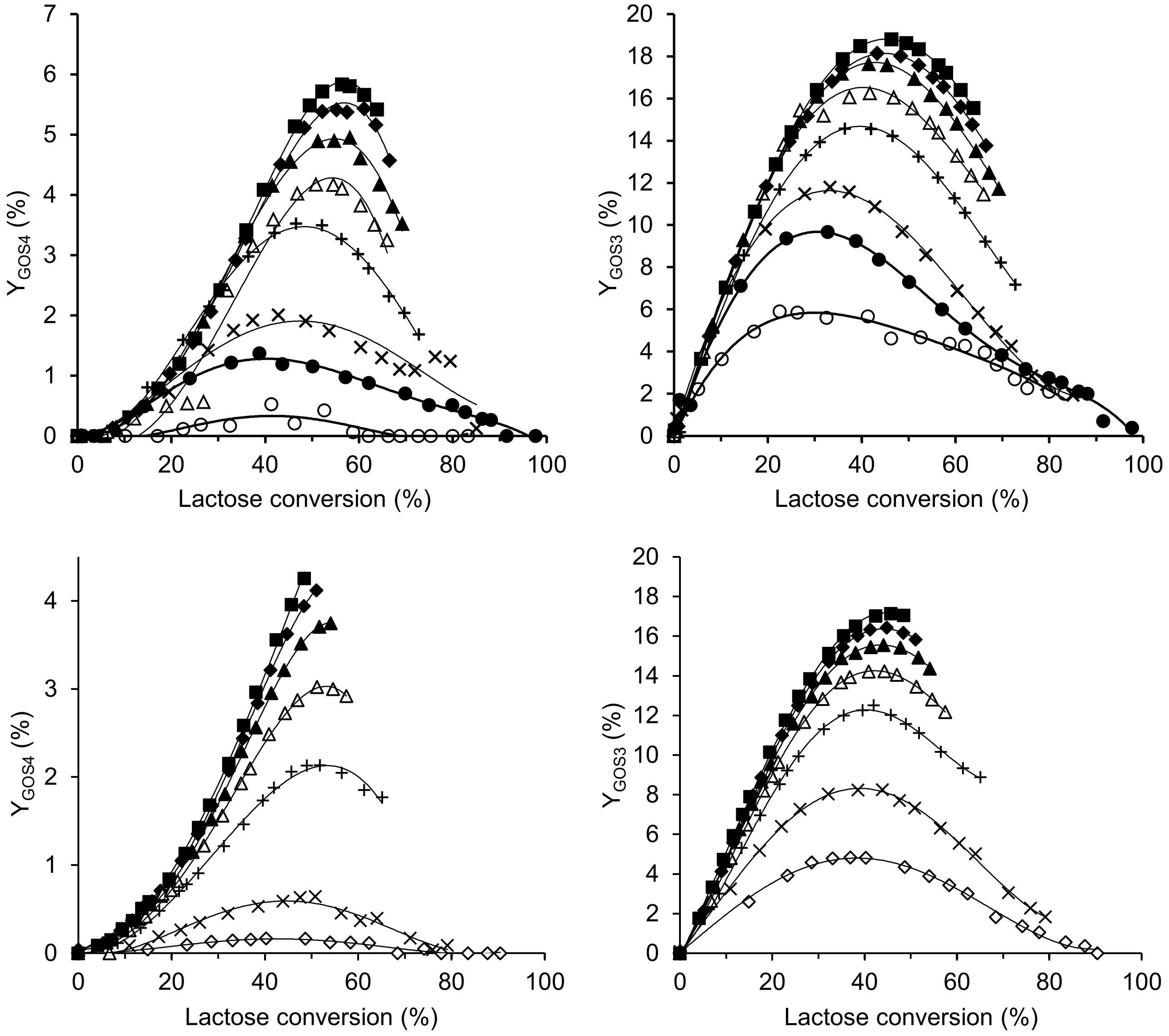

Figure S3. Effect of initial lactose concentration on yields of GOS4 and GOS3 catalyzed by free β-galactosidase (upper graphs), or immobilized biocatalyst CAT2 (lower graphs). Initial lactose concentrations: ■ 300 g/L, ◆ 250 g/L, ▲ 200 g/L, △ 150 g/L, + 100 g/L,× 50 g/L, ● 30 g g/L, ◇ 25 g/L, and ○10 g/L. Reaction conditions: temperature 42°C, enzyme concentration 16.7 U/mL (free enzyme) and 4.5 U/mL (CAT2).

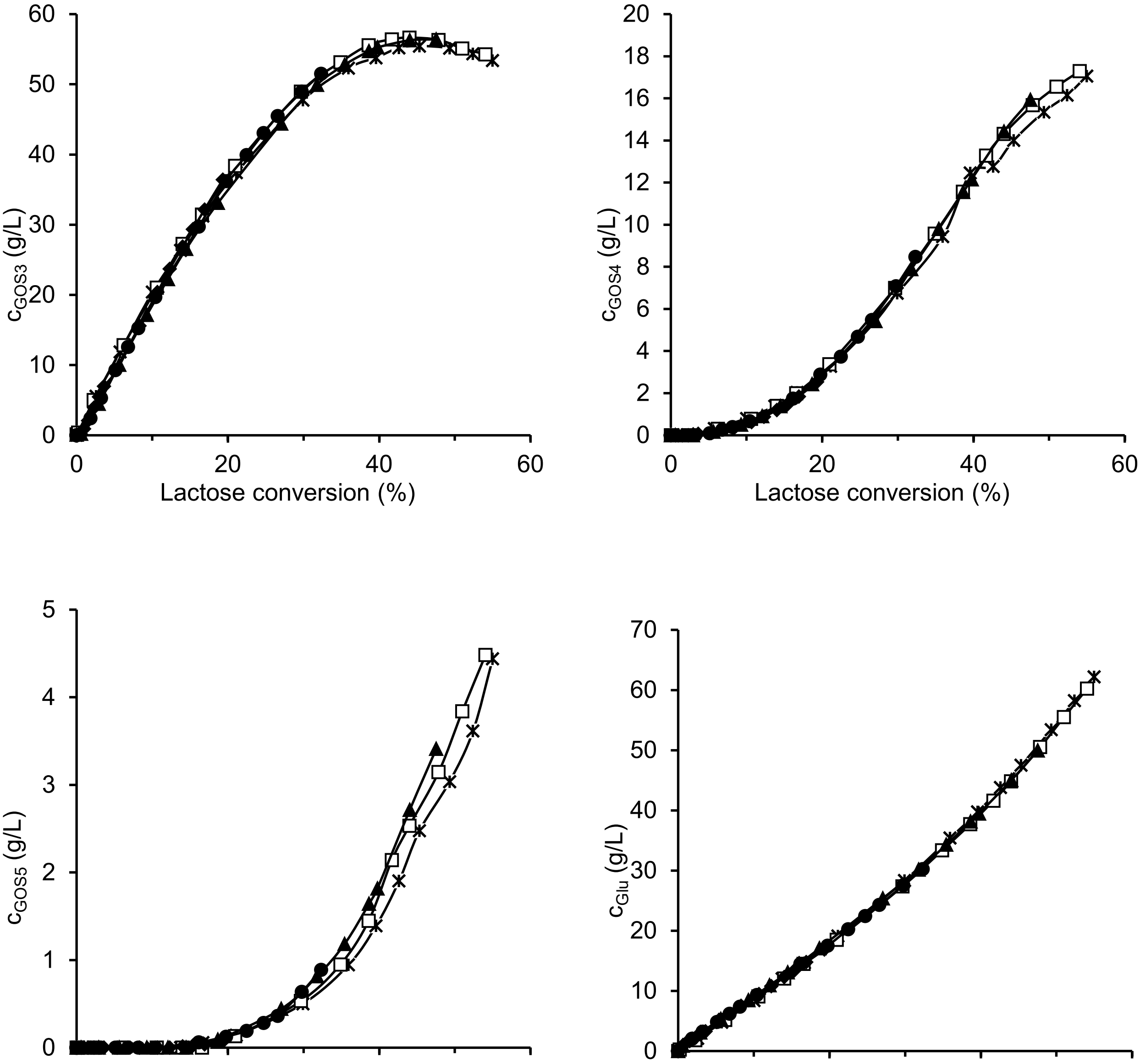

Figure S4. Effect of pH on the concentration of individual saccharides. Symbols: ✳ pH 4, □ pH 5, ▲ pH 6, ● pH 7, and ◆ pH 8. Reaction conditions: initial lactose concentration 300 g/L, enzyme concentration 16.6 U/mL (free enzyme), temperature 42 °C.

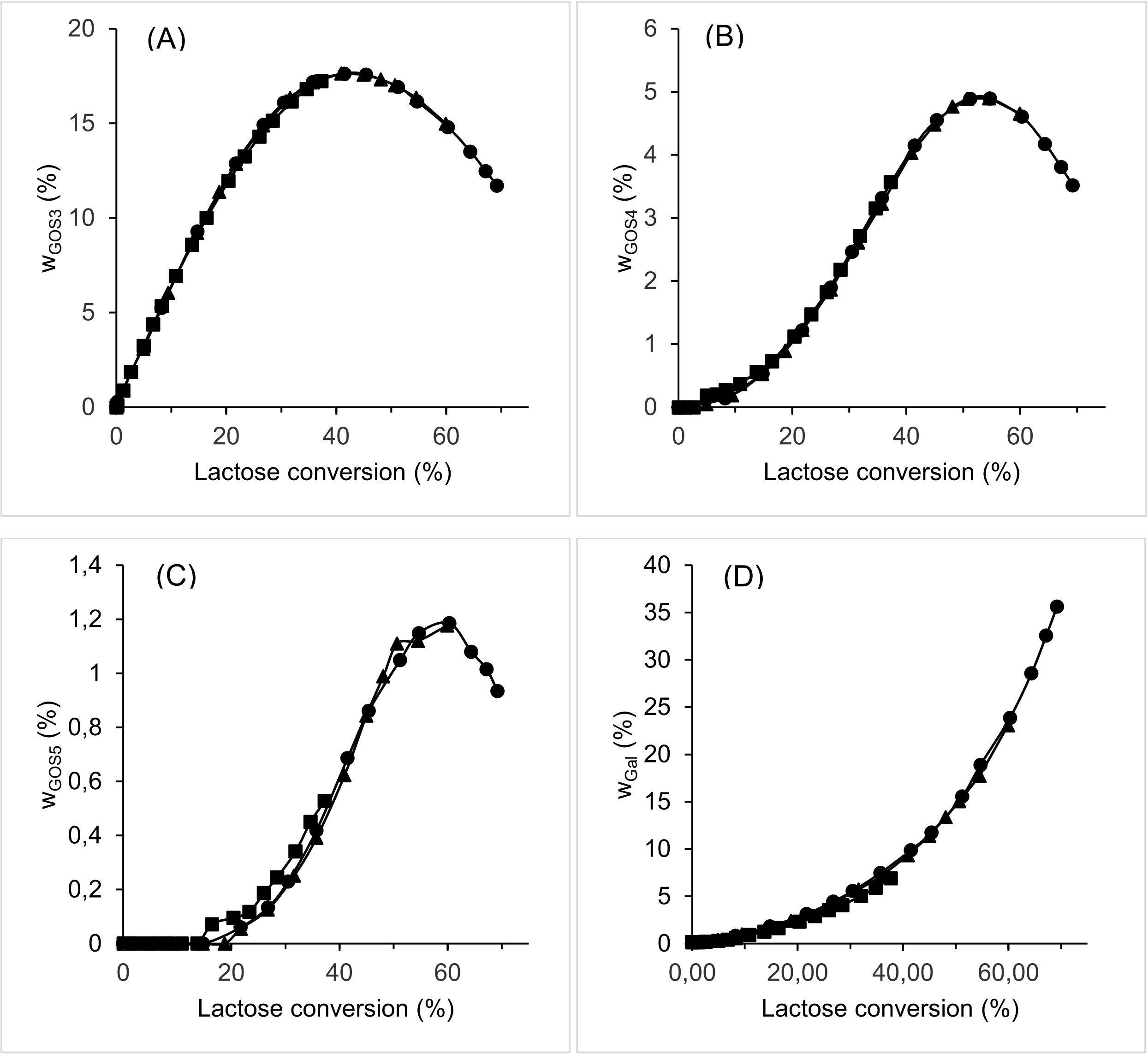

Figure S5. Effect of enzyme concentration on the composition of GOS mixture, expressed as the percentage of individual saccharide in total GOS (% w/w): (A) GOS3; (B) GOS4; (C) GOS5; and (D) galactose. Reaction conditions: initial lactose concentration 200 g/L, pH 4.5, temperature 42 °C, free enzyme concentrations: ■ 3.3 U/mL, ▲ 16.7 U/mL, and ● 33.3 U/mL.

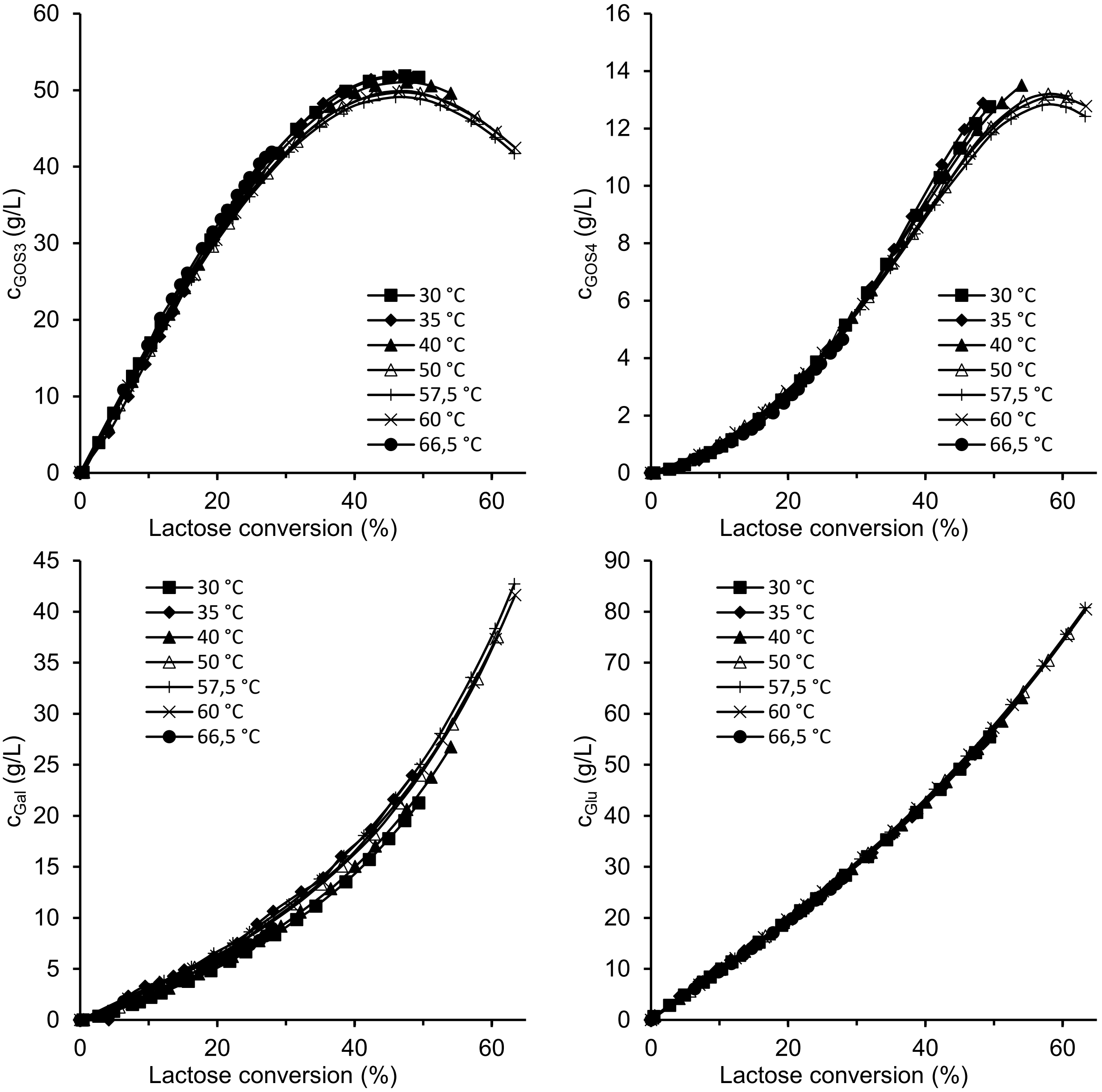

Figure S6. Effect of temperature on saccharide production by immobilized biocatalyst CAT2. (A) Time dependence of GOS concentration, and (B) dependence of GOS yield on lactose conversion. Reaction conditions: initial lactose concentration 300 g/L, enzyme concentration 4 U/mL, pH 4.5, temperatures: ■ 30 °C, ◆ 35 °C,▲ 40 °C, △ 50 °C,+ 57.5 °C,× 60 °C, and ● 66.5 °C.

Table S1 Effect of initial lactose concentration on lactose conversion, GOS yield, and mass fraction of individual GOS in total GOS (w/w) at maximum GOS concentration. Reaction conditions: free enzyme concentration 33.3 U/mL, temperature 42 °C, pH 4.5. X_{max} is the lactose conversion at maximum GOS concentration, c_{GOS}^{max}.

c_{S0} g/l	X_{max} %	c_{GOS}^{max} g/L	Y_{GOS}^{max} %	w_{GOS3} %	w_{GOS4} %	w_{GOS5} %	w_{Glb} %
10	29.4	0.6	5.1	95.1	4.9	0	0
30	33.3	3.0	10.6	90.1	9.2	0.5	0
50	36.8	7.0	13.9	83.3	13.7	1.6	1.4
100	46.6	19.3	20.1	70.7	17.5	4.2	7.6
150	54.5	33.3	23.1	64.2	18.0	6.3	10.4
200	54.6	49.9	24.7	64.5	19.9	5.1	9.8
250	55.3	66.9	26.5	64.7	20.5	4.9	9.5
300	56.8	84.7	28.0	62.7	21.1	5.7	9.7

Table S2 Effect of initial lactose concentration on lactose conversion, GOS yield, and mass fraction of individual GOS in total GOS (w/w) at maximum GOS concentration. Reaction conditions: biocatalyst CAT2 loading 50 mg/mL, temperature 42 °C, pH 4.5. X_{max} is the lactose conversion at maximum GOS concentration, c_{GOS}^{max}.

c_{S0} g/L	X_{max} %	c_{GOS}^{max} g/L	Y_{GOS}^{max} %	w_{GOS3} %	w_{GOS4} %	w_{GOS5} %	w_{Glb} %
25	37.0	1.3	5.3	96.9	3.1	0	0
50	43.9	4.5	10.5	93.3	6.7	0	0
100	45.7	14.8	14.6	82.2	14.1	1.4	2.3
150	47.0	27.3	185.0	77.9	16.0	1.9	4.3
200	51.7	41.8	20.6	72.3	18.0	3.0	6.8
250	51.1	53.8	21.3	74.3	19.3	3.4	2.9
300*	54.0	75.4	24.9	65.8	17.9	5.0	11.3

* maximum was not reached during 8 hours
